# Natures Transformations

**Published:** 1889-11

**Authors:** 


### NATURES TRANSFORMATIONS.

There are many wonderful tranformations in nature besides those which are common to the eye of the ordinary observer. Take for example the Medusa or common jelly fish. Born of the sea, the young hydrozoon becomes primarily, a distinct free germ, resembling a grain of rice, next a fixed cup having four lips, which subsequently turn to tentacles, and it becomes a hyatine flower which presently splits across the calyx into segments, whereupon it is made to resemblo a pine cone crowned with a tuft of transparent filament. Now the cone changes into a series of sea-daises threaded on a pearly stock, and these one by one become detached and float away, each a perfect little Medusa with purple bill and trailing tentacles.

Sterling Baking Powder.—In these days of conscienceless food adulteration it is a pleasure to be able to recommend to our patrons a pure article of Baking Powder, as our chemist assures us the Sterling is. Ask your grocer for it.

